# Evaluation of a Multisectoral Health Security Alliance Program Through Perceptions of Member States: African Partnership Outbreak Response Alliance (APORA)

**DOI:** 10.1093/milmed/usae125

**Published:** 2024-05-08

**Authors:** Amber J Rollings, Elizabeth Pertner, Haley Bockhorn, Jessica L A Jackson, Danny Shiau

**Affiliations:** Center for Global Health Engagement, Uniformed Services University for Health Sciences, Bethesda, MD 20817, USA; Henry Jackson Foundation for the Advancement of Military Medicine, Inc., Bethesda, MD 20817, USA; Center for Global Health Engagement, Uniformed Services University for Health Sciences, Bethesda, MD 20817, USA; Henry Jackson Foundation for the Advancement of Military Medicine, Inc., Bethesda, MD 20817, USA; Center for Global Health Engagement, Uniformed Services University for Health Sciences, Bethesda, MD 20817, USA; Henry Jackson Foundation for the Advancement of Military Medicine, Inc., Bethesda, MD 20817, USA; Center for Global Health Engagement, Uniformed Services University for Health Sciences, Bethesda, MD 20817, USA; Henry Jackson Foundation for the Advancement of Military Medicine, Inc., Bethesda, MD 20817, USA; Center for Global Health Engagement, Uniformed Services University for Health Sciences, Bethesda, MD 20817, USA

## Abstract

**Introduction:**

U.S. DoD global health engagements offer opportunities for strategic engagement and building capability in collaboration with foreign military and civilian counterparts. Global health engagement activities can take the form of health security alliances and allow the USA and its allies and partners to prepare for, mitigate, and respond to emerging biothreats and other harmful health events that may negatively impact national security. One such example is the African Partnership Outbreak Response Alliance (APORA), which was designed to expand African Partner Nation militaries’ infectious disease outbreak response capabilities. This publication evaluates the development, implementation, and outcomes of APORA to better understand the program’s effectiveness in developing Partner Nation medical capabilities and the efficacy of health security alliances more broadly.

**Materials and Methods:**

Key informant interviews, focus groups, and questionnaires were used to collect responses from a sample of participants who attended an in-person APORA event in May 2022. The research team conducted thematic analysis of all responses to identify common themes and sub-themes in participants’ perspectives and to elucidate findings and recommendations for future endeavors.

**Results:**

The analysis determined that participants attended the APORA event primarily to disseminate and apply knowledge, skills, and abilities gained at the event to their own health system structures. Overall, participants indicated that APORA contributed to their countries’ military medical and civilian cooperation, as well as their countries’ military medical capabilities. Longer-term partners (i.e., 4+ years of APORA membership) agreed more strongly with these sentiments; newer partners (i.e., 1-3 years of APORA membership) were more likely to be neutral or agree to some extent. Participants also valued the opportunity to solidify global, regional, local, and peer partnerships and considered the ability to create partnerships of great importance to their countries’ national health security. Language barriers were often listed as a hindrance to event participation and the overall integration of a regional health system response. Participants also cited resource scarcity, network erosion (particularly because of the coronavirus disease 2019 pandemic), and a lack of disseminating and communicating value-add in how APORA could/is providing to their member countries’ health systems as key barriers.

**Conclusions:**

As a whole, these findings support APORA’s objectives to develop and leverage partnerships to support medical capacity building, promote collaboration between military and civilian sectors, and increase access to opportunities and financial resources. Further evaluation is required to capture additional civilian perspectives while continuing to expand upon military perspectives in order to produce more generalizable findings. That said, this study enables key stakeholders to understand how to strengthen and expand future alliances to improve both health and security outcomes.

## INTRODUCTION

Health security alliances have become an essential element of global health security in response to known and emerging health threats that affect economies, politics, and societies.^1–4^ Multisectoral collaboration fostered by health security alliances enables diverse sectors to prepare for, mitigate, and respond to these threats. In particular, civilian–military partnerships offer unique opportunities for strategic engagement with multisectoral alliances.

The U.S. DoD has a significant stake in promoting civilian–military partnerships to aid in force health protection and promote resilient security environments. The African Partnership Outbreak Response Alliance (APORA) is one such example of a health security alliance developed by the U.S. DoD through U.S. Africa Command (USAFRICOM) and U.S. Air Forces in Europe and Air Forces Africa. In response to the 2014 Western African Ebola virus disease (EVD) epidemic, the USAFRICOM Surgeon’s Office initiated APORA to assist regional countries in strengthening prevention, detection, and response capabilities.^5,6^ The alliance uses coordinated engagement across African Partner Nations’ (PN) military and civilian health sectors to collectively prevent and respond to infectious disease outbreaks. Following the EVD epidemic, African PNs continued to effectively deploy military medical capabilities to combat infectious disease outbreaks including coronavirus disease 2019 (COVID-19), with responsibilities ranging from expanding testing capacity to assisting with vaccination drives.^7^

APORA is chartered as “an AFRICOM facilitated, African-led initiative to address the African PNs militaries’ capability to effectively respond to a disease outbreak and assist with Military Support to Civilian Authorities in disease outbreaks.”^8^ Central to this statement are the military–military relationships between USAFRICOM and the Ministries of Defense (MoDs) of PNs and the military–civilian partnerships between PN MoDs and their respective Departments or Ministries of Health (MoHs).

APORA MoD and MoH members present health system expertise at biannual meetings. APORA meetings typically span several days and include representatives from African PN MoDs and MoHs, intergovernmental organizations such as the United Nations and the African Union, and U.S. government agencies including CDC, United States Agency for International Development (USAID), USAFRICOM, U.S. Air Forces in Europe and Air Forces Africa, as well as programs such as the National Guard State Partnership Program.

In May 2022, APORA hosted CDC instructors for an in-person Rapid Response Team (RRT) Workshop in Accra, Ghana. During this workshop, researchers from the USU Center for Global Health Engagement (CGHE) were invited to collect data on the development, implementation, and outcomes related to APORA. These findings helped to illuminate opportunities and barriers for U.S. DoD participation in multisectoral health alliances.

## METHODS

### Data Collection

Approval for this research was obtained by USU’s Institutional Review Board (Protocol DBS.2022.320 [Reference No. 946135]), January 20, 2022. Convenience and snowball sampling were used to recruit participants for semi-structured key informant interviews (KIIs), focus group discussions (FGDs), and questionnaires at the RRT Workshop held in Accra, Ghana, in May 2022. USU CGHE researchers worked with workshop organizers to disseminate information on the study and opportunities to participate to attendees.

Potential participants self-selected into the study by indicating interest on a sign-up sheet maintained by the research team. Participants for either KIIs or FGDs were accepted on a first-come, first-serve basis. Interview participation was open to attendees with all levels of interaction with APORA, including first-time attendees, implementers, and APORA leadership. Interviews occurred in a quiet, private location and lasted 15 to 50 minutes. Informed consent was obtained both verbally and in writing for all interview participants. When necessary, the research team used translators to ensure accuracy in conducting interviews.

Interviews were conducted with a semi-structured protocol that included questions on a range of topics including military–civilian cooperation in the PN’s country, reasons for APORA participation, APORA’s contributions to cooperation, capabilities, and partnerships, as well as challenges to participation and recommendations for the program. Before the event, the research team used cognitive interviewing and pilot testing with relevant subject matter experts to ensure the appropriateness, relevance, and validity of the interview instrument used (Supplementary Tool Protocol 1).

Interviews were conducted using either a KII or FGD format with both modes utilizing the same protocol. The interview team comprised 2 experienced researchers from CGHE (A.J.R. and E.P.) with expertise in interviewing, evaluation theory and practices, and research methods. Interviews were recorded, translated when necessary, and transcribed verbatim. The research team reviewed all transcripts for accuracy and removed potentially identifying information.

A paper questionnaire (in English and French) was also distributed during the final session of the 2022 APORA event (Supplementary Tool Protocol 2). The questionnaire’s content followed similar themes as the interview protocol. As there were a limited number of interview sessions, the questionnaire was utilized to obtain additional data on PN perceptions and supplement interview data. Individuals who participated in an interview could also complete a questionnaire (Supplementary Table S1). Data describing attendance history, length of membership, and attitudes toward APORA were drawn from questionnaire responses; interviews were used to inform thematic analysis for this study.

### Analytic Approach

Content and thematic analysis was conducted on qualitative data. Thematic analysis is a method by which patterns of similar perspectives, sentiments, or issues are identified across multiple data sources and synthesized into common themes.^9^ Content analysis was guided by an a priori codebook with domains based on the interview and focus group protocols. After the first wave of coding, the team refined the codebook to eliminate unnecessary codes, identify patterns in responses, and meaningfully group patterns using thematic analysis. Between each of these iterations, coders applied inter-rater reliability to ensure each coder was utilizing the codebook in a sufficiently reliable way. The final inter-rater reliability calculation yielded a Cohen’s kappa of 74% agreement. All qualitative analysis was conducted using NVivo qualitative data analysis software (v12, Lumivero, Denver, CO).

The team reviewed identified themes to elucidate emergent sub-themes, findings, and recommendations. Descriptive statistics were produced for closed-ended items on the questionnaire using Excel (2019, Microsoft Corporation, Redmond, WA). Open-ended questionnaire items were analyzed qualitatively in conjunction with interview transcripts, using the same codebook.

## RESULTS

### Interviews

In total, 21 semi-structured interviews were conducted using either a KII (*n* = 18) or FGD (*n* = 3) format to collect information about participants’ perspectives on military–civilian medical coordination and APORA (Table I). All FGDs comprised 2 participants. Twenty-seven percent of APORA PN representatives in attendance at the event participated in KIIs (*n* = 15) or FGDs (*n* = 1), with the majority (*n* = 88%) being PN MoD affiliates.

**TABLE I.** Participating Stakeholder KIIs and FGDs

Participating stakeholder	Key informant interview	Focus group discussion
Partner Nation participants: Algeria, Cameroon, Cape Verde, Chad, Côte d’Ivoire, Gabon, Ghana, Guinea, Kenya, Liberia, Niger (FGD), Senegal, South Africa, Uganda	15	1
APORA Advisors and Observers: U.S. interagency representatives	2	0
U.S. DoD Champions: APORA facilitators and implementers	1	2
Total	18	3

This table presents the composition of stakeholder participation in KIIs and FGDs. Fifteen KIIs and 1 FGD were conducted with MoH and/or MoD representatives from 14 APORA member PNs. Additionally, 3 KIIs were conducted with 2 U.S. interagency representatives (Advisors and Observers) and an event implementer (U.S. DoD Champion), and 2 FGDs occurred with event facilitators (U.S. DoD Champions). All FGDs comprised 2 respondents.

Abbreviations: APORA, African Partnership Outbreak Response Alliance; FGDs, focus group discussions; KIIs, key informant interviews; MoD, Ministry of Defense; MoH, Ministry of Health; PNs, Partner Nations.

### Questionnaires

A total of 48 paper-based questionnaires were collected out of 64 PN representatives in attendance, with an overall response rate of 75% (Table II). PN MoD representatives comprised 81% of questionnaire respondents (*n* = 39) and 19% identified as PN MoH representatives (*n* = 9).

**TABLE II.** PN Study Participants by Affiliation and Reviewed by Country’s Length of APORA Membership and Participants’ Event Attendance

Sector affiliation by PN APORA membership and event attendance	Ministry of Defense		Ministry of Health	
	Number of MoD respondents	Percentage of MoD respondents	Number of MoH respondents	Percentage of MoH respondents
Questionnaire responses (*n* = 48)	39	81%	9	19%
Length of country’s APORA membership				
New (1-3 years)	10	26%	3	33%
Mid-level (4-6 years)	8	20%	2	22%
Long-term/founding (7+ years)	21	54%	4	45%
APORA event attendance history				
First time	24	62%	7	78%
2-4 times	9	23%	2	22%
5+ times	6	15%	0	0%

This table describes the number of PN participants from either the MoD or MoH by length of their PN’s APORA membership and their event attendance history; only participants who completed a questionnaire are described.

Abbreviations: APORA, African Partnership Outbreak Response Alliance; MoD, Ministry of Defense; MoH, Ministry of Health; PN, Partner Nation.

Though more MoH attendees were from countries with long-term or founding-level (7+ years) APORA membership at 45% (*n* = 4), representation for newer (1-3 years) and mid-level (4-6 years) APORA membership was fairly similar by count (*n* = 3 and *n* = 2, respectively). In contrast, more than twice as many MoD representatives from long-term or founding members attended (*n* = 21), compared to MoD attendees from newly joined (*n* = 10) or mid-level members (*n* = 8). For both sectors, the majority of attendees represented long-term or founding-level countries, yet the majority of questionnaire respondents were first-time event attendees (65% of total responses, *n* = 31). Seventy-eight percent of MoH representatives (*n* = 7) and 62% of MoD representatives (*n* = 24) were attending for the first time. All respondents who had attended an APORA event 5 or more times were MoD representatives.

Of note, questionnaire respondents represented 23 APORA member countries (Algeria, Angola, Benin, Cameroon, Cape Verde, Chad, Côte d’Ivoire, Democratic Republic of the Congo, Gabon, Ghana, Guinea, Kenya, Liberia, Madagascar, Morocco, Niger, Senegal, Sierra Leone, South Africa, Tanzania, Togo, Tunisia, and Uganda). Ghana had the most responses (*n* = 7), followed by Cameroon (*n* = 3) and Côte d’Ivoire (*n* = 3) (Supplementary Table S1). The majority of PNs provided both an interview and a questionnaire response.

### Reasons for Attending APORA Events

**FIGURE 1.**
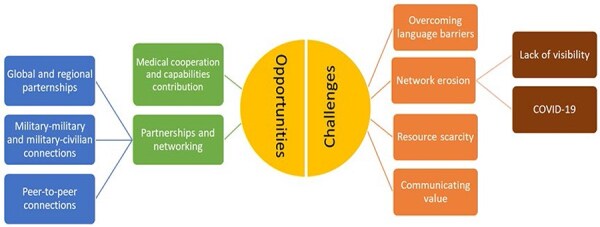
Respondents’ perceptions of opportunities and challenges from participating in the African Partnership Outbreak Response Alliance (APORA). This figure depicts themes and sub-themes of opportunities and challenges from participating in the APORA described by study participants in questionnaire responses, key informant interviews, and focus group discussions.

In interviews and in response to the questionnaire’s open-ended questions, study participants noted their intentions to disseminate and apply the knowledge, skills, and abilities gained from the event. A number of attendees further mentioned their intention of training colleagues when back in-country, highlighting further sustainment of the workshop’s learning applications. Additionally, a number of respondents highlighted a desire for standardization of methods, such as through standard operating procedure (SOP) development. Although this particular topic may have been brought up more often because of a CDC training at the APORA event focusing on RRTs and SOPs, its utility is important to note as study participants considered development and implementation of SOPs to be instrumental in their countries’ ability to prevent, detect, and respond to infectious disease outbreaks. Respondents also discussed how attending the events exposes them to ideas for improvements to their health system structures as they had the opportunity to learn from other attendees’ experiences and best practices. Findings from the questionnaires and interviews further elucidated the opportunities and challenges that arose from participation in APORA (Fig. 1).

### Opportunities Enabled by APORA Participation

#### Opportunity 1: Medical cooperation and capabilities contribution

**FIGURE 2.**
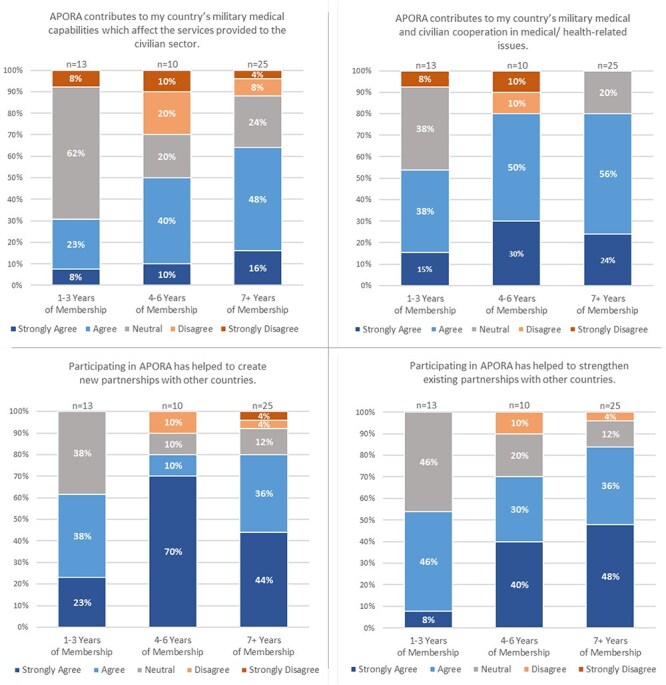
Partner Nation agreement by membership length. This figure depicts Partner Nation participants’ sentiments on the African Partnership Outbreak Response Alliance (APORA).

When asked to reflect on APORA’s current or continuing contributions, the majority of questionnaire respondents agreed or strongly agreed that participating in APORA strengthens military–civilian medical cooperation (73%) and builds medical capabilities (53%) (Supplementary Table S2). Specifically, questionnaire participants from PNs with 4 or more years of membership agreed most that APORA contributed to their country’s military medical and civilian “cooperation” in medical and/or health-related issues (80% of mid-level and long-term respondents agreed or strongly agreed) (Fig. 2). One key informant described APORA’s impact on military–civilian cooperation in their country:

So, I guess what [APORA] also did was to allow us, for instance, in the [PN] armed forces, to engage well with the national authorities […] So, that’s a whole thematic area that deals with that collaboration. Security and civilian health authorities. And we tried to fashion out particular rules for security agencies, the military, the police, etc., and how they could also augment the national response. So, I guess APORA heightened our awareness of the broader issues of the - made us aware of the linkage, the importance to link ourselves more into the national programs. (PN, KII)

Additionally, questionnaire respondents from long-term or founding PNs (7+ years) were more likely to agree that APORA contributed to their country’s military medical “capabilities” (64% agree or strongly agree), while 50% of the respondents from mid-level (4-6 years) member PNs agreed to some degree with the statement. However, less than half of the respondents from newly joined (1-3 years) APORA PN members agreed to some extent with this statement (31%) and demonstrated the most neutrality (62%) of all membership lengths (Fig. 2).

When asked to reflect on how APORA has contributed to their country’s medical capabilities, one interviewee mentioned how APORA facilitated connections with experts in incident management, which greatly assisted in emergency operation center management during the COVID-19 pandemic. Interview participants also mentioned early detection, epidemic control, capacity building, fundraising, strategy development, resilience, and developing SOPs as benefits of membership. While topically informative, further discussion or explanation of APORA’s role in affecting these more tangible benefits was limited.

#### Opportunity 2: Partnerships and networking

Survey respondents were asked if APORA participation contributed to partnership development, particularly regarding relationships between regional partners and military and civilian agencies. Sub-themes identified from responses include global and regional partnerships; military–military and military–civilian connections; and peer-to-peer connections.

The majority of questionnaire respondents affirmatively responded that APORA helped to create (75%) and strengthen (73%) partnerships (Supplementary Table S2). New and mid-level members agreed more that APORA created new partnerships (61% and 80% agreed or strongly agreed, respectively) than strengthened partnerships (54% and 70% agreed or strongly agreed, respectively). Long-term PNs were more likely to agree or strongly agree that APORA both strengthened partnerships (84%) and created partnerships (80%), showing high levels of agreement overall toward these effects (Fig. 2).

Key informants also noted that creating and maintaining global and regional partnerships are of great importance to their national health security. As one interviewee commented:

…we’re living in a globalizing world. In disasters, emergencies, diseases; they know no borders, so we’ve got to be part of the world. (PN, KII)

Key informants also discussed how APORA has strengthened regional coordination. Regional relationships are key, as participants noted that having the support of surrounding countries as well as providing support to those countries is an integral part of their continent’s unity. APORA was described as an important avenue for different militaries to connect, particularly during the 2014 EVD epidemic. Interviewees affirmed APORA provided new access among military medical experts in different countries. Additionally, APORA supported greater communication between military and civilian medical partners.

APORA will really help them so that there will be both a civil and a military collaboration to tackle epidemics. (PN, KII)

Questionnaire and interview respondents also noted that networking opportunities at in-person events provided the added benefit of peer-to-peer learning, such as document sharing and information exchanges. Attendees were eager to learn from each other and anticipated drawing on their peers’ lessons learned upon returning to their own countries. Overall, participants found the APORA event as a valuable opportunity to solidify global, regional, local, and peer relationships.

### Challenges of APORA Participation

#### Challenge 1: Language barriers

Language barriers were listed as hindrances to the integration of a regional health system response, as well as for full participation during the APORA event. One respondent noted:

…our groups alone cannot be matched…it has to be Anglophone versus Francophone, versus the Portuguese speaking…So, we’re not able to integrate. So, then that makes me wonder, how do you share ideas? And then, how do you do an alliance? How do you respond to things? We have to find a common language. (PN, KII)

#### Challenge 2: Network erosion

Connecting with others and collaborating within APORA was found to be difficult because of network erosion, where social connectivity was weak or absent. Decreased connection hampered stakeholder investment and participant engagement. Two prominent sub-themes for network erosion for APORA participants were lack of visibility and the COVID-19 pandemic. Lack of visibility pertained to both military and civilian sectors where participants noted their colleagues’ unawareness of APORA:

Well, I see from other nations that they have involved the civilian arm. We haven’t. I don’t even know whether our civilian arm knows about APORA. (PN, KII)

Limits to in-person networking because of COVID-19 also presented significant challenges as participants felt that virtual environments stunted opportunities for engagement.

So, in the core concept of presentations you probably couldn’t articulate much of a difference. A presentation is a presentation. Seeing it virtual versus seeing it in person probably didn’t make much of a difference. The level of participation and engagement is completely different. So, there weren’t really opportunities that were conducive opportunities for question and answers. We kind of did, but it really wasn’t the same. We didn’t get people to actually ask a question. (U.S. DoD Champions, FGD)

#### Challenge 3: Resource scarcity

For some countries, even sponsored event attendance can be costly as their country’s public health infrastructure cannot afford the loss of human capital for the length of the event or similar engagements that would keep them from their daily duties and responsibilities.

It’s a huge, huge issue to pull their medical personnel from their regions because of how limited it is and the fact that they may be the only medical personnel in that area, that region. (U.S. DoD Champion, FGD)

#### Challenge 4: Communicating value

Another key challenge of APORA participation is communicating the value-add of APORA, particularly regarding knowledge dissemination through engagements like training workshops. Difficulty implementing ideas or methodologies also minimizes APORA’s value-add, as highlighted by another PN respondent:

So, APORA, personally I got to know APORA because my boss used to come for all the APORA conferences. However, nothing much grew out of it, I think, in my view. You attend, and that will be that. I don’t think you have established anything with us. I’m not sure…So, as far as APORA is concerned, honestly, I’ve not seen what collaboration we’ve had with them. But then, what I’m seeing from this conference is that there’s a lot we could gain. (PN, KII)

## DISCUSSION

This study explores the benefits, challenges, and overall impact of APORA membership on PN health systems, military medical capabilities, and military–civilian medical cooperation. Active participation in APORA engagements helps member states align their health security goals with one another as well as with broader global health security frameworks.^10–13^ These overlaps help to systematize strengthening public health systems for global prevention, detection, and response to infectious disease.

The significance participants placed on learning from other PNs at APORA events highlights how membership enables PNs to improve their military–civilian medical cooperation and capabilities. PNs with longer APORA membership had greater agreement on APORA’s contributions to their countries. This, along with the emphasis attendees placed on knowledge sharing at APORA events, suggests that APORA is successfully carrying out its objective of identifying best practices from African partners and sharing those best practices among members.^14^

Respondents’ high agreement levels regarding APORA’s ability to create and strengthen partnerships supports their charter objective to utilize current partners and mobilize new partners and additional financial resources for pandemic programs for the military services.^14^ When countries participate in APORA, they find value in the ability to leverage APORA’s international, regional, national, and local connections to build influence and support for their health systems, encouraging robust and resilient health sectors. As such, the DoD’s role as a stakeholder is crucial as it is uniquely positioned to help PNs build these civilian–military alliances in ways that other actors (e.g., intergovernmental organizations) are not able to support.

Participants frequently cited language barriers and network erosion as challenges for both APORA engagements and larger health system responses among PNs. The breakdown of language barriers needs to be deliberate and focused appropriately to ensure expertise and knowledge is being shared as best as possible. To combat network erosion and for better posturing of APORA to occur, information about the benefits of APORA participation should be more effectively communicated at the PN level, such as intraministerially and intersectorally.

These increased communications, especially within the civilian sector, may help to boost visibility and encourage buy-in from newer member countries, who demonstrated the most neutrality when asked if APORA contributed to creating and strengthening parternships (38% of participants from newly joined member countries were neutral when asked if APORA helped to create new partnerships with other countries, compared to 10% of participants from countries with mid-level APORA membership and 12% of participants from countries with long-term APORA membership; 46% of participants from newly joined member countries were neutral when asked if APORA helped to strengthen existing partnerships with other countries, compared to 20% participants from countries with mid-level APORA membership and 12% of participants from countries with long-term APORA membership (Fig. 2). The contrast between newer and more experienced members may evidence the impact of engagement type (virtual versus in-person) on relationship building. Participants from newer member countries are likely to have engaged in primarily virtual events because of COVID-19 limitations, whereas participants from mid-level and longer-term member countries are more likely to have participated in virtual and in-person events.

Understanding potential barriers to participation is significant, as nearly all respondents, regardless of their countries’ membership length, placed a high value on relationship building. Hosting more in-person APORA events may mitigate network erosion and encourage participation more broadly. That said, these in-person events may place a strain on existing country health system support. As previously indicated, some countries cannot afford the absence of personnel during the training events and further considerations will need to be made to encourage participation across all PNs.

Respondents also identified communicating the value-add of APORA as another challenge of maintaining interest and investment in alliance. PN participants appreciated receiving training; however, difficulties implementing the knowledge and skills learned during the training led participants to question tangible benefits. These challenges may be mitigated as APORA shifts toward more active, applied engagements. Changes in the broader global health security environment may increase the visibility of APORA’s value-add as well, as there has been wider recognition of the utility of multisectoral alliances like APORA following the COVID-19 epidemic.

Despite ongoing challenges, APORA has influenced member PNs’ public health security capacities by increasing access to technical training, knowledge-sharing platforms, and enhanced networking through interoperable and multisector engagements. Furthermore, APORA contributes to the advancement of technical skills, standardization of policies and procedures, and the development and strengthening of multilateral partnerships. Partnerships such as these could enable public health systems to better address a wider array of future social and economic disruptors particularly in the African region, such as stewardship and resourcing to reduce the high level of antimicrobial resistance burden.^15^

Based on the findings of this report, the recommendations are as follows: (1) maintain technical training and activities, such as drafting and adopting multinational SOPs for joint health crisis response, to increase a more tangible value-add to PN stakeholders; (2) conduct and evaluate multinational simulation exercises to apply the training of members and disseminate best practices and lessons learned; (3) provide additional translation resources to increase networking opportunities at events; and (4) expand PN MoH and civilian participation in the program. Future evaluation studies should further clarify how military–civilian relationship building translates into changes in medical capabilities and increase the purview of the civilian sector while continuing to expand upon the military sector’s perspectives.

### Limitations

There are limitations to consider in this evaluation study, specifically regarding data collection and the study population. Data collection was limited by time constraints at the event; interviews took place during the RRT Workshop, posing a scheduling conflict for attendees who wished to participate in the study. Interviews were understandably a lower priority for attendees, and increased schedule flexibility could have enabled more in-depth discussions, as well as more time for follow-up with respondents to expand on information provided.

The study population posed another limitation, as more MoD participants completed interviews and questionnaires than MoH participants. Although this was reflective of the RRT Workshop population, it limited how much information could be gleaned from the civilian perspective regarding military–civilian collaboration. PN representation in the study population should also be considered as a limitation, as most countries included in the study were represented by only 1 or 2 respondents (Supplementary Table S1). This may limit the generalizability of findings, especially at the country level.

In addition, the majority of attendees at the workshop had not previously attended an APORA event. First-time attendees may not be closely familiar with APORA’s objectives or the program’s longitudinal impact on their country’s health system. What is more, the high proportion of first-time attendees points to possible issues in maintaining continuity of training, as workshops can be designed to build off of previous training or events. Finally, it is important to acknowledge the potential impacts of social-desirability bias; participants may have chosen to highlight positive contributions and minimize critical reflections to ensure that APORA continues to be supported by the U.S. DoD.

## CONCLUSION

The purpose of a health security alliance is to strengthen and expand security preparedness by leveraging multisectoral health partnerships to provide support across diverse sectors and communities. The study findings suggest that APORA promotes collaboration between military and civilian sectors, allowing for cohesive knowledge building and foundational partnering opportunities. Additionally, APORA provides training to PN MoD and MoH personnel and acts as a platform for subject matter experts to expand their networks, increasing workforce capital in the military and civilian sectors. Maintaining and improving health systems capable of protecting public health requires a skilled workforce; as such, APORA’s contributions to multisectoral partnerships add strategic value and resiliency to public health systems and global health security.

## Supplementary Material

usae125_Supp

## Data Availability

The authors confirm that the data supporting the findings of this study are available within the article and/or its supplementary materials.
