# Supplementary material for: Evaluation of a Multisectoral Health Security Alliance Program Through Perceptions of Member States: African Partnership Outbreak Response Alliance (APORA)
Source: Mil Med. 2024 May 8;190(1-2):324–32. doi: 10.1093/milmed/usae125 (PMC11737319; doi:10.1093/milmed/usae125)
Supplement: usae125_Supp [file usae125_supp.zip › supp/APORA_Supplementary_Table_S1.docx]

Supplementary Table 1. Details of Data Types Collected by APORA Member Country

| APORA Member Country | KII/FGD | Questionnaire Participants | | Data Collection Total per Country |
| --- | --- | --- | --- | --- |
|  |  | MoD Respondents | MoH  Respondents |  |
| Algeria | 1 | 0 | 1 | 2 |
| Angola | 0 | 1 | 1 | 2 |
| Benin | 0 | 1 | 0 | 1 |
| Burkina Faso* | 0 | 0 | 0 | 0 |
| Cameroon | 2 | 3 | 0 | 5 |
| Cape Verde | 1 | 1 | 0 | 2 |
| Chad | 1 | 2 | 0 | 3 |
| Côte d’Ivoire | 1 | 2 | 1 | 4 |
| Democratic Republic of the Congo | 0 | 1 | 1 | 2 |
| Gabon | 1 | 1 | 0 | 2 |
| Ghana | 1 | 7 | 0 | 8 |
| Guinea | 1 | 1 | 1 | 3 |
| Kenya | 1 | 2 | 0 | 3 |
| Liberia | 1 | 1 | 1 | 3 |
| Madagascar | 0 | 2 | 0 | 2 |
| Mali* | 0 | 0 | 0 | 0 |
| Morocco | 0 | 2 | 0 | 2 |
| Niger | 1 | 1 | 1 | 3 |
| Nigeria* | 0 | 0 | 0 | 0 |
| Rwanda* | 0 | 0 | 0 | 0 |
| Senegal | 2 | 2 | 0 | 4 |
| Sierra Leone | 0 | 1 | 1 | 2 |
| South Africa | 1 | 1 | 0 | 2 |
| Tanzania | 0 | 2 | 0 | 2 |
| Togo | 0 | 1 | 1 | 2 |
| Tunisia | 0 | 2 | 0 | 2 |

Supplementary Table 1 (continued).

| Uganda | 1 | 2 | 0 | 3 |
| --- | --- | --- | --- | --- |
| Total | 16 | 39 | 9 | 64 |

Table S1 details the data collected via the key informant interviews (KIIs), focus group discussions (FGDs), and questionnaire responses. Individuals who participated in a KII/FGD could also complete a questionnaire. All Partner Nations (PNs) in attendance at the Workshop participated via the KII/FGDs, questionnaire, or both. In addition, the table lists the African Partnership Outbreak Response Alliance (APORA) member countries which did not attend the Workshop, and thus were not able to be included in the survey - *Burkina Faso, *Mali, *Nigeria, *Rwanda.
