# Supplementary material for: Evaluation of a Multisectoral Health Security Alliance Program Through Perceptions of Member States: African Partnership Outbreak Response Alliance (APORA)
Source: Mil Med. 2024 May 8;190(1-2):324–32. doi: 10.1093/milmed/usae125 (PMC11737319; doi:10.1093/milmed/usae125)
Supplement: usae125_Supp [file usae125_supp.zip › supp/APORA_Supplementary_Table_S2.docx]

Supplementary Table 2. Questionnaire Outcomes for APORA Member Countries

| Country’s length of APORA membership (Years) | Strongly Agree | Agree | Neutral | Disagree | Strongly Disagree | Totals |
| --- | --- | --- | --- | --- | --- | --- |
| **APORA contributes to my country’s military medical and civilian cooperation in medical/ health-related issues.** | | | | | | |
| 1 - 3 | 2 | 5 | 5 | 0 | 1 | 13 |
|  | 15% | 38% | 38% | 0% | 8% |  |
| 4 - 6 | 3 | 5 | 0 | 1 | 1 | 10 |
|  | 30% | 50% | 0% | 10% | 10% |  |
| 7+ | 6 | 14 | 5 | 0 | 0 | 25 |
|  | 24% | 56% | 20% | 0% | 0% |  |
| Totals | 11 | 24 | 10 | 1 | 2 | 48 |
|  | 23% | 50% | 21% | 2% | 4% |  |
| **APORA contributes to my country’s military medical capabilities which affect the services provided to the civilian sector.** | | | | | | |
| 1 - 3 | 1 | 3 | 8 | 0 | 1 | 13 |
|  | 8% | 23% | 62% | 0% | 8% |  |
| 4 - 6 | 1 | 4 | 2 | 2 | 1 | 10 |
|  | 10% | 40% | 20% | 20% | 10% |  |
| 7+ | 4 | 12 | 6 | 2 | 1 | 25 |
|  | 16% | 48% | 24% | 8% | 4% |  |
| Totals | 6 | 19 | 16 | 4 | 3 | 48 |
|  | 13% | 40% | 33% | 8% | 6% |  |
| **Participating in APORA has helped to create new partnerships with other countries.** | | | | | | |
| 1 - 3 | 3 | 5 | 5 | 0 | 0 | 13 |
|  | 23% | 38% | 38% | 0% | 0% |  |
| 4 - 6 | 7 | 1 | 1 | 1 | 0 | 10 |
|  | 70% | 10% | 10% | 10% | 0% |  |
| 7+ | 11 | 9 | 3 | 1 | 1 | 25 |
|  | 44% | 36% | 12% | 4% | 4% |  |
| Totals | 21 | 15 | 9 | 2 | 1 | 48 |
|  | 44% | 31% | 19% | 4% | 2% |  |
| **Participating in APORA has helped to strengthen existing partnerships with other countries.** | | | | | | |
| 1 - 3 | 1 | 6 | 6 | 0 | 0 | 13 |
|  | 8% | 46% | 46% | 0% | 0% |  |
| 4 - 6 | 4 | 3 | 2 | 1 | 0 | 10 |
|  | 40% | 30% | 20% | 10% | 0% |  |
| 7+ | 12 | 9 | 3 | 1 | 0 | 25 |
|  | 48% | 36% | 12% | 4% | 0% |  |
| Totals | 17 | 18 | 11 | 2 | 0 | 48 |
|  | 35% | 38% | 23% | 4% | 0% |  |

Table S2 covers the results for the descriptive statistics analysis for the questionnaire items on the African Partnership Outbreak Response Alliance (APORA) affecting military-civilian cooperation and medical capabilities, and creating and strengthening partnerships. Respondents’ perception levels are arranged by their affiliated country’s APORA membership length.
