# Supplementary material for: Evaluation of a Multisectoral Health Security Alliance Program Through Perceptions of Member States: African Partnership Outbreak Response Alliance (APORA)
Source: Mil Med. 2024 May 8;190(1-2):324–32. doi: 10.1093/milmed/usae125 (PMC11737319; doi:10.1093/milmed/usae125)
Supplement: usae125_Supp [file usae125_supp.zip › supp/APORA_Supplementary_ToolProtocol_S1.docx]

Supplementary Tool Protocol 1

Key Informant Interview/Focus Group Discussion (KII/FGD) Semi-Structured Interview Protocol

**Semi-Structured Interview Protocol for Partner Nation (PN) Leaders**

Critical questions are bolded below. If constrained by time or other obstacles, please prioritize asking critical questions.

1. **How would you describe your role at your organization?**

1. **If MoH, are you affiliated with the Ministry of Defense?**

☐ Yes ☐ No

1. **If MoD, are you affiliated, enlisted, or commissioned in the military?**

☐ Affiliated ☐ Enlisted ☐ Commissioned

1. **If *in* the military - What is your rank and branch of service?**

1. **What is your primary work location?**

=============================================================

1. **How does the military support the civilian population in medical or health-related activities in [Partner Nation]?**
   1. **For example, does the military include medical personnel that deliver care to civilians? (if this clearly answered in #5, skip to 5.2)**
   2. **Does the military have an official role in providing humanitarian services, relief, or medical care directly to civilians? (if this clearly answered in #5, skip to 5.3)**
   3. Are there any other ways the military supports civilian health? (for example, points of entry inspection, etc.)
   4. **Is there a formalized process or document directing any of these activities? (Examples might also include an organization chart, line diagram, or graphical representation.)**
2. Is this support a full-time assignment or limited to emergency declarations, crisis response, or specific medical missions?
3. **Do military medical providers play a role at civilian medical facilities?**
   1. **Are these military medical staffing requirements formally identified? (prompt: formalized through documents? Other processes?)**
   2. **Are there expectations for the amount and type of care delivered by military medical providers to civilians? (prompt: this may include direct patient care to civilians and/or support to non-defense ministries or authorities)**
      1. Can you please describe and quantify if possible the care provided to civilians delivered by military health providers? (i.e. percentage of civilians treated on a daily/monthly basis and type of care delivered)
      2. [If quantification is unknown, ask if they have any suggestions on who might have that information]
4. Are there any restrictions on military medical support for civilians?
   1. If so, where are these restrictions recorded? (law, doctrine, etc.)
5. **What role do you have in relation to APORA?**

1. How long have you been involved with APORA?

1. How many APORA events have you attended?

1. What specific APORA events did you engage in and when?

1. **What are the reasons for [Partner Nation] to participate in APORA?**
2. **Can you provide an example of when military medical and civilian cooperation on medical or health-related issues has been successful?**
   1. **Is this example an activity that has been able to be maintained? (prompt: why or why not?)**
   2. **Has participating in APORA contributed positively or negatively to this in any way?**
3. **Can you provide an example of when military medical and civilian cooperation on medical or health-related issues has faced challenges?**
   1. **Were these challenges overcome? How?**
   2. **Has participating in APORA contributed positively or negatively to this in any way? (prompt: request examples/specifics)**
4. Has [your organization] developed any new partnerships or strengthened any existing partnerships with other organizations, or other countries, as a result of participating in APORA? [If no, skip]
   1. Will you describe the partnerships? [prompt: how formalized are the partnerships?]
   2. How will these partnerships contribute to achieving [your organization's] medical or health-related goals?
   3. Could you share a few of your organization’s accomplishments achieved through these partnerships?
5. **As a result of participating in APORA, has your organization participated in programs, lines of effort, or activities that it had not participated in before?**
6. **Has your organization developed any new or changed any policies or standard operating procedures as a result of participation in APORA? (prompt: Have there been any changes in policy, practices, or funding, etc.)**
   1. **Has anything helped in applying these policies?**
   2. **Has anything hindered applying these policies?**
7. What have been the most beneficial aspects of participating in APORA for [Partner Nation]?
   1. Any particular metrics tracked for these impacts (either for management of APORA participation or for demonstrating impact?)
8. What is still needed from APORA to help reach [Partner Nation’s] shared health security goals?
   1. What is still needed to help [Partner Nation] **reach any goals** related to participating in APORA? (prompt: Resources, funding, personnel, policy, more training, better communication, etc.)
9. **Is there anything else you would like to share about APORA and/or other military activities with your civilian sector before we end the interview? (prompt: Request 1 - 2 other alliance/partner organizations we could potentially interview and/or shareable information (AARs, etc.))**
