# Supplementary material for: Evaluation of a Multisectoral Health Security Alliance Program Through Perceptions of Member States: African Partnership Outbreak Response Alliance (APORA)
Source: Mil Med. 2024 May 8;190(1-2):324–32. doi: 10.1093/milmed/usae125 (PMC11737319; doi:10.1093/milmed/usae125)
Supplement: usae125_Supp [file usae125_supp.zip › supp/APORA_Supplementary_ToolProtocol_S2.docx]

Supplementary Tool Protocol 2

Questionnaire – English version

1. Which country are you representing at the APORA Conference? ___________________________
2. In which sector do you work? Ministry of Health Ministry of Defense/Military Other:

1. How many APORA events have you attended? First time 2 – 4 times 5+ times
2. Are you an Executive Board member of APORA? Yes No

**General questions: Please respond by circling in each row your level of agreement to each statement.**

| ***Rate your level of agreement with each statement*** | Strongly Agree | Agree | Neutral | Disagree | Strongly Disagree |
| --- | --- | --- | --- | --- | --- |
| 1. The military supports the civilian population in medical activities in my country. | 5 | 4 | 3 | 2 | 1 |
| 1. Military medical providers play a role at civilian facilities in my country. | 5 | 4 | 3 | 2 | 1 |
| 1. Medical military facilities and services are accessible to the civilian population. | 5 | 4 | 3 | 2 | 1 |
| 1. Medical military facilities and services are the only type of care accessible to the civilian population. | 5 | 4 | 3 | 2 | 1 |

**APORA related questions: Please respond by circling in each row your level of agreement to each statement. *Please note: If you are a first time attendee, then respond to the “potential” of how well you think these statements could fit for your country.***

| ***Rate your level of agreement with each statement*** | Strongly Agree | Agree | Neutral | Disagree | Strongly Disagree |
| --- | --- | --- | --- | --- | --- |
| 1. APORA contributes to my country’s military medical and civilian cooperation in medical/ health-related issues. | 5 | 4 | 3 | 2 | 1 |
| 1. APORA contributes to my country’s military medical capabilities which affect the services provided to the civilian sector. | 5 | 4 | 3 | 2 | 1 |
| 1. Participating in APORA has helped to create new partnerships with other countries. | 5 | 4 | 3 | 2 | 1 |
| 1. Participating in APORA has helped to strengthen existing partnerships with other countries. | 5 | 4 | 3 | 2 | 1 |
| 1. Participating in APORA has provided an opportunity to improve my country’s overall health-related capabilities. | 5 | 4 | 3 | 2 | 1 |

1. What laws or regulations govern your military medical activities for the civilian sector?
2. How can APORA help provide support to your country’s military medical and civilian cooperation on medical or health-related issues? (Please use the backside of this page for responding if needed.)
